# A Golden Age of Brain Exploration

**DOI:** 10.1371/journal.pbio.0030024

**Published:** 2005-01-18

**Authors:** Virginia Gewin

## Abstract

The Allen Brain Atlas of gene expression in the mouse brain is poised to serve as an outstanding resource to neuroscience

Armed with billions of cells, elaborate circuitry, and a seemingly animate anatomy, capable of growing as it learns, the brain is a marvelously enigmatic organ. Much to the chagrin of those that study it, the brain remains perhaps too mysterious.

Although genetic information exploded out of the Human Genome Project, it has been of little consequence to neuroscience—a discipline still grappling with the boundaries and names for distinct brain regions. According to United States National Institute of Mental Health Director Thomas Insel, over 99% of the neuroscience literature focuses on only 1% of the estimated 15,000–16,000 genes expressed in the brain. David Van Essen, a neurobiologist at Washington University in St. Louis, Missouri, likens the current genetic map of the brain to a 17th century map of Earth. A voyage around the Earth had already proven it was round, but landmass resolution was still vague at best.

Magellan's benefactors, though, never bankrolled a technical advance quite like the Allen Brain Atlas. Neuroscience's unlikely sugar daddy, Microsoft cofounder and the world's fifth wealthiest man, Paul Allen, created the $100 million dollar Allen Brain Institute in Seattle, Washington, two years ago. The first explicit goal of the institute was to create an open-access, visual, searchable online map of genes expressed in the brain, as well as of brain circuitry and cell location. Roughly one petabyte of data—equal to the memory necessary to hold the information held in about 50 Libraries of Congress—will be produced as a result.

In mid-December, the first 2,000 genes were uploaded. By 2006, the Allen team plans to have as many as 24,000 genes online. While the ultimate goal is to map the human brain, the atlas ushers in a new era of neurogenetics—an attempt to make connections between anatomical, genetic, and behavioral observations.

## Of Mice and Men

The initial effort will focus on the standard, inbred lab mouse strain known affectionately as C57BL6. Like it or not, mice are remarkably similar to humans—sharing 99% of our genes. Humans, at this time, provide too many hurdles—not the least of which is a lack of willing brain donors that are the same age. Since the C57BL6 strain is inbred, the mice are also much more uniform than humans—a key to constructing the most accurate representative map possible of one species' adult brain.[Fig pbio-0030024-g001]

**Figure pbio-0030024-g001:**
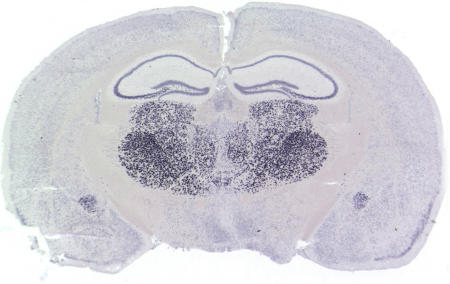
In situ hybridization—A cross-section through the mouse brain shows gene expression (black dots) in specific brain regions (Copyright: David Anderson)

With a map of mouse genes in hand, scientists will be able to develop informed hypotheses about genes that may affect human brain function and dysfunction. “People will be able to look first in the mouse atlas, then more selectively focus on human cases,” says Gregor Eichele, director of the Max Planck Institute for Experimental Endocrinology in Hannover, Germany. Indeed, researchers may do well to focus their efforts on those specific cells in which homologous genes are expressed in the mouse. Insel suggests an initial mental health application: find *dyspyndin*, a gene linked to schizophrenia. Insel is banking on the atlas to locate genes linked to conditions including bipolar disorder, schizophrenia, and autism. Once identified, such critical genes can be examined in detail and used in studies aimed at disease cures or drug screens. Presumably, the atlas will be a boon for drug discovery and development by providing information on drug targets present within brain cells.

Eichele points out that the Allen Brain Atlas will eliminate time wasted testing fruitless hypotheses. “If you think gene X may be involved in [hippocampal-dependent] memory, but it's not expressed in the hippocampus, you shouldn't bother following that line of research,” he says. Insel agrees that where genes are expressed in the brain will be most telling. “In the brain, more than any other organ, function follows form,” he says.

Cellular resolution of expression patterns will prove necessary to uncover as yet unknown relationships between circuitry, cell type, and gene expression in the brain, says Arthur Toga, a neuroscientist at the University of California, Los Angeles, and Allen Brain Atlas advisor. Ed Lein, a neuroscientist at the Allen Brain Institute, thinks that mapping at the cellular scale will also redefine anatomy. Traditionally, neuroanatomists have delineated brain regions pretty much by eye, identifying clusters of cells and patterns of connections that look the same. “We're starting to redefine boundaries of regions by cell type,” says Lein, defining cell type by gene expression pattern.[Fig pbio-0030024-g002]

**Figure pbio-0030024-g002:**
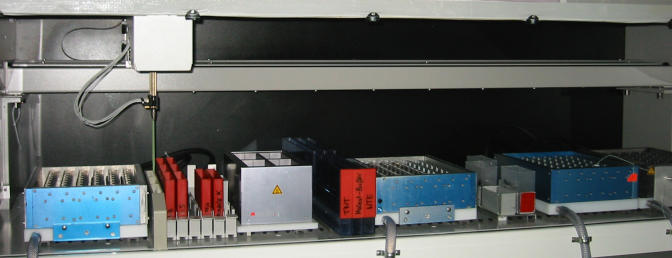
“Robot” used for high-throughput in situ hybridization, developed by Gregor Eichele and colleagues at the Max Planck Institute for Experimental Endocrinology (Photo: Gregor Eichele)

The difficulty, according to Allen Brain Institute scientific advisor David Anderson, a neuroscientist at the California Institute of Technology in Pasadena, California, is in understanding how a gene mutation affects behavior. “It is impossible without a knowledge of the circuits in which a gene is expressed,” he says. Anderson himself studies innate behaviors such as fear responses. Specifically, he's most interested in understanding the function of different regions of the almond-shaped amygdala. Once he finds genes expressed in neurons within regions of the amgydala implicated in fear, he plans to determine neuron function by creating transgenic animals in which specific neuron activity has been silenced.

Thus far, Anderson's laboratory has endured the slog of using microarray techniques to identify genes with expression patterns linked to fear behavior, followed by in situ hybridization, which traditionally involves twenty-odd complex, error-prone steps, to find just some of the genes expressed in different parts of the amygdala. “The Allen Brain Atlas will identify a whole set of genes that we would have had to spend several years to find,” he says. Indeed, locating where even one gene of interest is expressed in the brain eats up valuable research time, especially when there are so many potentially interesting genes. In most cells, 10,000 genes can be expressed.

Eichele improved the slow error-prone process into an automated, fast, “high throughput” method that caught the attention of the Allen team because it was capable of meeting the needs of such an ambitious project. In situ hybridization uses labeled probes for specific messenger RNA sequences, allowing scientists to test individual brain tissue samples for gene expression.

Automated in situ hybridization data will be generated for the entire mouse transcriptome—the full complement of activated genes in a particular tissue at a particular time—on a genome-wide scale. The mouse is a young adult at 56 days old, free from the confounding factors of development. After it is sacrificed, the mouse brain is immediately frozen, then sliced very thinly—to get forty sections from each millimeter of thickness—so that the probes for hybridization can expose gene expression in individual cells.

## Mining the Mind for Riches

The in situ data will be matched in a three-dimensional framework to the reference atlas developed by Allen's team. The resulting images will be turned into a virtual microscope, allowing users to focus down on genes expressed in regions of interest.

While the Allen Brain Atlas is somewhat like other genomics projects in scale, it is unique. “No one's gone into a 3-D structure like a tissue and examined it in a systematic way,” says Allan Jones, senior director of Allen Brain Atlas Operations. In that way, he adds, it's a much richer dataset than the Human Genome Project. “As we're ramping up—fully by spring of next year—we'll be generating about 1,000 microscope slides a day with four mouse brain sections on each slide,” says Jones. Each day, those sections are scanned and stitched together electronically into 300-megabyte batches. “Scaling up a lab process is currently the biggest challenge,” says Jones. “In effect, we're turning an art form into something that gives high-quality data day in and day out,” says Jones. “If you are off slightly when cutting a 2-D brain slice, it becomes very difficult to map back into a 3-D context.”

While it is a logical starting point, a spatial understanding of gene expression is just one way to mine the brain. Other avenues currently being pursued explore individual variability, development, and comparisons between species (See Box 1). The sheer comprehensive nature of the Allen Brain Atlas will be its crowing achievement, and its complement to the other ongoing atlas efforts. However, the magnitude of the project also poses its greatest hurdle—about which onlookers have expressed some concern.

**Box 1. A Bevy of Brain Databases**

The Allen database will provide a spatial map of neurogenetic data, specifying where the 99% of shared mammalian genes are expressed in the brain. Other online databases are striving to provide alternative axes of information that will detail individual variability, species comparisons, and changes during development.

Going deep on the genetic variability axis of understanding is Web QTL, a collection of images from 800 brains of 35 different strains of mice. “We're interested in genetic sources of variation,” says Rob Williams, a neurobiologist at the University of Tennessee in Memphis. “We study many strains of mice and map the upstream modulators that control expression differences.”

Van Essen's online atlas strives to map the structural and functional areas of the cerebral cortex, believed to be the seat of thought, learning, emotion, sensation, and movement, for humans, macaques, rats, and mice. In constructing the SuMS (for “surface management system”) database, they've put most of their effort into comparing datasets between species.

Finally, GenSAT (Gene Expression Nervous System Atlas) follows gene expression as it changes through the development of an organism. Using a method that manipulates “bacterial artificial chromosomes” to insert, change, or delete parts of large gene sequences, one transgenic mouse is created for each gene. When reporter genes are added to the bacterial artificial chromosomes, cells with selective gene activity glow. This advance takes a step towards relating gene expression patterns to connectivity between brain regions.[Fig pbio-0030024-g003]

“It's clear that they want to do a first-rate job of working with all this information, but I'm not sure they entirely appreciate how hard it's going to be to manage the staggering amount of information they're getting,” says Van Essen. As manager of his own comparative anatomy database, he understands the magnitude of the task. He points out that properly digitizing the data in electronic form and registering one particular slice to a standardized reference with meaningful coordinates is not trivial.

“In an atlas, there will be considerable variability from one individual to the next—even in inbred mouse strains,” says Van Essen. Even if it's only 20% variability, it is still going to pose challenges for managing experimental data. Van Essen acknowledges that this isn't just an impediment for Allen's team, but for neuroscience as a whole. “These tools, in general, don't emerge from a vacuum. They emerge best when rich, challenging datasets are staring people in the face,” he says.

An even larger problem is communicating the data effectively. Figuring out how to navigate the tremendous morass of data will be a bioinformatic stumbling block. Lein acknowledges that figuring out how to best annotate the data is one of the bigger challenges for the future—particularly in cases where gene expression doesn't match the agreed-upon boundaries of anatomical regions.

For now, users will be able to mine the data by gene name only. The initial release consists of an image viewer to view the in situ hybridization data for one or several genes at a time, along with a reference atlas to determine the structures in which genes are expressed. Future releases will allow the user to conduct more sophisticated searches, such as by anatomical structures.

“Not only is there this 3-D structure, but there are lots of studies where people are trying to understand what drives the turning on and off of genes,” says Jones. “At the end, if the atlas has a big impact, it will be in providing the precise coordinates for those people to tease apart what specific DNA elements drive expression within regions or structures.”

And while the Allen Brain Atlas will provide a fine level of detail, there are limitations. Much like the Human Genome Project, the information will be the starting point and not the end point of understanding brain function. “It won't change strategy for doing experiments,” says Nobel laureate and Columbia University neuroscientist Eric Kandel. “The atlas will be a catalyst rather than a direction setter.”

## The Final Frontier

Overall, neuroscience is entering a new era. Insel notes that recent work has proven the brain to be extraordinarily dynamic, birthing neurons throughout a lifespan. Brain functions seem more modular than global. And there is no real separation between the mind and brain. “Mental disorders are brain disorders,” he says.

Over the next 5–10 years, neurogenomics will fuel a golden age of discovery in neuroscience. In fact, scientists may even reach an overarching goal—understanding how the wild card of environment impacts brain function. “We want predictive genetics able to accommodate environmental differences,” says Rob Williams, neurobiologist at the University of Tennessee in Memphis.

For Kandel, one thing is certain. “Most of the mysteries of the brain lie ahead of us.”

**Figure pbio-0030024-g003:**
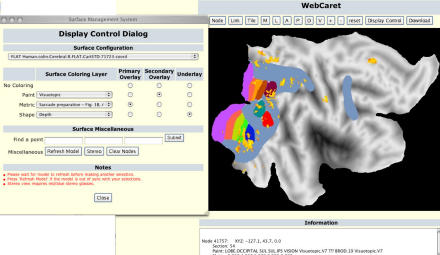
Using WebCaret and SumsDB to visualize functional magnetic resonance imaging activations and visual areas on a flat map of the human “Colin” cortical atlas (Image: David Van Essen; dataset with publication sources available: http://sumsdb.wustl.edu:8081/sums/directory.do?dir_id=702541)
